# Micro-computed tomography: Introducing new dimensions to taxonomy

**DOI:** 10.3897/zookeys.263.4261

**Published:** 2013-02-04

**Authors:** Sarah Faulwetter, Aikaterini Vasileiadou, Michail Kouratoras, Thanos Dailianis, Christos Arvanitidis

**Affiliations:** 1 Department of Zoology-Marine Biology, Faculty of Biology, National and Kapodestrian University of Athens, Panepistimiopolis, 15784, Athens, Greece,; 2 Institute for Marine Biology, Biotechnology and Aquaculture, Hellenic Centre for Marine Research, 71003 Heraklion, Crete, Greece; 3 Department of Biology, University of Patras, 26504, Rio, Patras, Greece; 4 Hellenic Centre for Marine Research, 71003 Heraklion, Crete, Greece

**Keywords:** Micro-computed tomography, systematics, taxonomy, 3D, visualisation, interactive PDF, polychaetes, cybertypes, cybertaxonomy

## Abstract

Continuous improvements in the resolution of three-dimensional imaging have led to an increased application of these techniques in conventional taxonomic research in recent years. Coupled with an ever increasing research effort in cybertaxonomy, three-dimensional imaging could give a boost to the development of virtual specimen collections, allowing rapid and simultaneous access to accurate virtual representations of type material. This paper explores the potential of micro-computed tomography (X-ray micro-tomography), a non-destructive three-dimensional imaging technique based on mapping X-ray attenuation in the scanned object, for supporting research in systematics and taxonomy. The subsequent use of these data as virtual type material, so-called “cybertypes”, and the creation of virtual collections lie at the core of this potential. Sample preparation, image acquisition, data processing and presentation of results are demonstrated using polychaetes (bristle worms), a representative taxon of macro-invertebrates, as a study object. Effects of the technique on the morphological, anatomical and molecular identity of the specimens are investigated. The paper evaluates the results and discusses the potential and the limitations of the technique for creating cybertypes. It also discusses the challenges that the community might face to establish virtual collections. Potential future applications of three-dimensional information in taxonomic research are outlined, including an outlook to new ways of producing, disseminating and publishing taxonomic information.

## Introduction

Morphology-based taxonomy has been at the heart of systematic research for over two centuries. Over the last decades, however, the dominant role of morphology in systematics and phylogenetics has been challenged by an increasing number of analyses supported by molecular data (Cook et al. 2010, Giribet 2010). Sequence data are being produced at a rapid speed and are readily available for constructing phylogenies or delimiting species. However, the formal description and naming of species and other biological units is still central to biodiversity research (Budd and Olsson 2007, Deans et al. 2011). This highly accelerated data acquisition creates an imbalance between availability of data and the human power to actually interpret them and thus to create new knowledge. In taxonomy, this “gap in scalability” (Giribet 2010) becomes even more problematic due to the time-consuming and still largely manual process of describing and naming new species which cannot keep up with the speed at which new information becomes available from the molecular world. As a consequence, a steadily increasing number of species are delimited genetically but lack a formal, morphology-based description (e.g. Audzijonyte et al. 2008, Barroso et al. 2009, Osborn and Rouse 2010). This problem is amplified by the so-called “taxonomic impediment”: fewer and fewer resources—both human and financial—are available for conventional taxonomic and systematic research (Carvalho et al. 2007), and the field is lacking a global electronic infrastructure (Godfray 2002a, Wheeler et al. 2004). The controversial debate over possible causes and remedies for the stagnation the discipline is experiencing (e.g. Knapp et al. 2002, Godfray 2002b, Carvalho et al. 2007, Evenhuis 2007, Joppa et al. 2011a, b) has stimulated governments and funding agencies to increasingly recognise its importance, and several encouraging developments have arisen over the last years. Besides releasing funds for training and education, much funding has been allocated to the field of cybertaxonomy, allowing the development of internet-based tools and resources aiming to boost taxonomic research and to accelerate the process of new species descriptions and systematic analyses. These developments include online resources such as name-based registers (e.g. Catalogue of Life – http://www.catalogueoflife.org , the Global Names Architecture – http://www.globalnames.org , ZooBank – http://www.zoobank.org , the World Register of Marine Species – http://www.marinespecies.org ), biogeographic databases (e.g. the Ocean Biogeographic Information System – http://www.iobis.org , the Global Biodiversity Information Facility – http://www.gbif.org ), aggregators and curators (e.g. Fishbase –http://www.fishbase.org , Encyclopedia of Life – http://www.eol.org ), virtual research environments targeted towards taxonomy (Scratchpads – http://www.scratchpads.eu ), increasing availability of literature, both for legacy literature (Biodiversity Heritage Library – http://www.biodiversitylibrary.org ) as well as an increase in open access literature and journals aiming at rapid publication of taxonomic treatments, including new publication models such as semantically enhanced information (Penev et al. 2010).

Despite the increase in information availability through these initiatives, one of the main bottlenecks in conventional taxonomy and systematics is still the availability of type material and thus reliable morphological information (Godfray 2007, Wheeler et al. 2012). Loans from museums are often difficult or even impossible to obtain and the time and effort to gather the material needed for a systematic revision can take months or years. The tediousness of this work contributes even further to the stagnation of the discipline: new data are produced at a slow pace, and this lack of new, readily accessible and computer-retrievable morphological data (Deans et al. 2011) prevents the testing of large-scale hypotheses, as they currently become common in phylogenomic analyses (Dunn et al. 2008, Philippe et al. 2009, Edgecombe et al. 2011). Godfray (2007) has argued that a possible solution to overcoming this bottleneck could be a web-based taxonomy using “cybertypes”, “based on the very best current imaging methods” (Godfray 2007), an idea immediately criticised by others (e.g Carvalho et al. 2007). The idea of creating virtual collections of taxonomic material is, however, indeed enticing, and first implementations of accurate imaging methods, mass digitisations and remote access to digital material have been recently presented in a dedicated collection of papers (Smith and Blagoderov 2012, and references therein). Technological advances and a new generation of imaging techniques will inevitably open new horizons not only by providing rapid access to first-hand morphological information but also by making this information accessible to humans and computers alike. Non-destructive three-dimensional imaging techniques such as confocal laser scanning microscopy (cLSM), optical projection tomography (OPT), magnetic resonance imaging (MRI) and micro-computed tomography (micro-CT), allow for rapid creation of high-resolution morphological and anatomical data in three dimensions (Giribet 2010, Ziegler et al. 2008, 2010, 2011a, for a detailed overview of the different techniques see e.g. Boistel et al. 2011, Laforsch et al. 2012). These techniques allow detailed virtual reconstructions of the morphology and anatomy of specimens and subsequent interactive manipulation (e.g. rotation, virtual dissection) and analysis of these data. Not only are they ideal for the digitisation of taxonomically important morphological information, but they allow new kinds of analyses (e.g. morphometrics in three dimensions) to be performed, thus creating novel directions of research. Indeed, the ability of these technologies to create three-dimensional, interactive models with a resolution in the micrometre scale or even below, combined with non-destructive sample assessment (as opposed to three-dimensional reconstruction of histological sections), has lately started to attract the attention of researchers beyond the traditional (clinical) applications of the methods, boosted by an increasing accessibility of micro-CT scanners and rapid computational advances. Particularly, invertebrate zoologists have started to employ micro-MRI (for an overview of taxa imaged so far with MRI see Ziegler et al. 2011a) and micro-CT. Several studies already show the potential of these methods to deliver new data to test taxonomic hypotheses (Heim and Nickel 2010, McPeek et al. 2011, Csösz 2012). They also provide new insights into morphology and anatomy (Golding and Jones 2006, Holford 2008, Dinley et al. 2010, Huckstorf and Wirkner 2011), functional morphology (Alba-Tercedor and Sánchez-Tocino 2011, Bond et al. 2008, Nickel et al. 2006, Patek et al. 2007, Wilhelm et al. 2011) and developmental studies (Postnov et al. 2002, Marxen et al. 2007, Puce et al. 2012) by studying species through a virtual, three-dimensional model. In palaeobiology, the technique is, for example, frequently used to reveal the morphology and even anatomy of fossilised organisms that cannot be removed from their enclosure medium (Dierick et al. 2007, Dunlop et al. 2011, Hendrickx et al. 2006, Molineux et al. 2007, Penney et al. 2007, Sutton 2008). Most of these studies have imaged few or a single specimens, but some have harnessed the power of non-invasive, three-dimensional imaging to create vast amounts of data for large-scale systematic analyses (Wirkner and Prendini 2007, Ziegler et al. 2008, Golding et al. 2009, Zimmermann et al. 2011).

Despite the increasing use of these new imaging methods, most of the recently created datasets might not qualify for the notion of a cybertype. In most studies, specimens were prepared and imaged with a specific hypothesis in mind, focusing on certain morphological characteristics and omitting others, and the resulting data might thus not be useful for other purposes. Datasets that are intended to serve as a cybertype should fulfil at least the following three basic assumptions: (a) A cybertype should provide morphological and anatomical information of the same accuracy and reliability as provided by the physical type material, independently of a specific research question in mind; (b) A cybertype should be linked to the original type material, which can be consulted if in doubt. This implies that any method used to create the cybertype should not affect the morphological, anatomical and molecular identity of the original specimen (e.g. holotype, paratype or neotype); (c) A cybertype has to be retrievable and freely accessible. This involves making the data available through a reliable (internet) source under an open access licence and providing adequate security measures, such as archiving, backups and ensuring data format compatibility in the future, and allowing the annotation of the dataset with metadata in order to be retrievable and interpretable.

Towards this end, this study explores the potential of micro-computed tomography to create high-throughput morphological and anatomical data to support systematic and taxonomic studies by using polychaetes (bristle worms) as a demonstration taxon for macro-invertebrates. This taxon has been chosen because of the diversity of shapes and tissue types occurring among its members, allowing the investigation of the behaviour of the methodology across a range of samples with different characteristics. The outcomes are evaluated with regard to the first requirement for constituting a potential cybertype, that is, their ability to deliver reliable information on diagnostic and systematically important characters. However, from sample preparation to the final presentation of the results many steps are involved which may affect both the outcome of the data as well as the original specimen. Particularly, the imaging of soft tissues with micro-CT might require tissue staining (Metscher 2009a, b), but neither the effects of contrast-enhancing chemicals nor of ionising radiation upon the integrity of tissue and genetic material are yet fully understood. Although micro-CT radiation seems to negatively affect the genetic material of living tissue (Wolff 1971, Kersemans et al. 2011), no fragmentation of the DNA could be detected in museum material of bird skins exposed to micro-CT scanning (Paredes et al. 2012). The morphological and molecular integrity of scanned material is particularly important when valuable museum material is imaged, otherwise the material is rendered useless for further investigations. Therefore, by testing whether treatment with contrast agents or exposure to X-ray radiation create structural damage to the tissue of the sample or impair the potential to amplify nucleic acid structures important for the molecular identification, this study assesses the compliance of micro-CT imaging with the second requirement for creating a cybertype. Finally, various aspects of exploring and communicating the resulting information through new ways of publishing are demonstrated and evaluated with regard to the third requirement for a cybertype. The paper concludes by summarising both the potential and the shortcomings of micro-CT imaging for taxonomic research and provides an outlook to possible future developments, including the overall applicability of the cybertype concept and the establishment of virtual collections.

## Material and methods

### Specimen preparation and processing

Nine polychaete specimens (seven different species) were chosen for this study, all of them in the clade Aciculata (Annelida, Polychaeta) (Table 1). Specimens are stored in the collections of the biodiversity laboratory of the Hellenic Centre for Marine Research, except for those of *Eunice roussaei* Quatrefages, 1866 (deposited in the Aristotelian University of Thessaloniki), *Alitta succinea* (Leuckart, 1847)and *Hermodice carunculata* (Pallas, 1776) (both subsequently used for molecular analyses and destroyed). All specimens had originally been fixed and preserved in different media, but were dehydrated to 96% ethanol prior to treatment. Identification was performed to the lowest possible level under a stereo microscope and light microscope, using the most recent literature available for each taxon (e.g. Böggemann 2002, San Martín 2003, Bakken 2004, Carrera-Parra 2006a, Zanol and Bettoso 2006). However, no dissections were performed, in order to assess whether internal characters required for identification in several species could be determined through virtual dissections instead.

**Table 1.** Overview of scanned polychaete specimens, their preparatory treatment and scanning parameters. † = maximum dimension (in any direction) of the scanned part of the specimen; ‡ = multi-part scan, automatically joined; § = distance between projection images in degrees; | = total size of cross-section images in megabyte, file format: PNG/BMP.

Species	Sample code	Location and depth	Size (mm) ^†^	Scanned part	Sample preparation	Scanning medium	Rotation step ^§^	Number of projection images	Exposure time per image (ms)	Total scanning time (h:min)	Resolution (μm per pixel)	Dataset size ^|^
*Lumbrineris latreilli* Audouin & Milne Edwards, 1834	ΦΗ20D	Gulf of Heraklion, Crete, Greece (35.3527°, 024.1084°), 20m	2.4	Anterior end	none	air	0.25°	1440	1925	3:08	1.919	506(BMP)
*Eunice* sp. (juvenile)	CALA-10b-08	Alykes, Crete, Greece (35.4158°, 024.9875°), 10m	1.8	Whole animal	24 h in iodine	96% ethanol	0.26°	1384	1835	3:33	2.162	69 (BMP)
*Eunice* sp. (juvenile)	CELB-10c-08	Elounda, Crete, Greece (35.2516°, 025.7583°), 1m	2.2	Whole animal	24 h in PTA	96% ethanol	0.3°	1200	1835	3:05	2.298	93(BMP)
*Eunice roussaei* Quatrefages, 1866	POL/EUN/503	Thermaikos Gulf, Greece	2.9	parapodium (mid-body)	2 h in HDMS	air	0.25°	1440	1155	2:19	2.878	326(BMP)
*Alitta succinea* (Leuckart, 1847)	F6BNR1	Etang de Berre, France (43.4699°, 005.1699°), 2m	8.1	Anterior end	20 days in PTA, solution renewed every 5 days	96% ethanol	0.4°	900	190	2 x 0:17 ^‡^	3.425	1433(PNG)
*Phyllodoce lineata* (Claparède, 1870)	Φ2Η25E	Gulf of Heraklion, Crete, Greece (35.3640°, 025.1086°), 25m	3.9	Anterior end	36 h in PTA	96% ethanol	0.28°	1285	1165	2:05	1.645	450(PNG)
*Phyllodoce* sp.	Φ2Η40D	Gulf of Heraklion, Crete, Greece (35.3832°, 025.1086°), 40m	5.5	Anterior end and mid-body	36 h in PTA	96% ethanol	0.28°	1285	1336	2:04	1.096	2406(PNG)
*Syllis gracilis* Grube, 1840	CELB-1a-07	Elounda, Crete, Greece (35.2516°, 025.7583°), 1m	7.6	Anterior end and mid-body	3 days in iodine	96% ethanol	0.25°	1440	1165	3 x 1:52 ^‡^	1.439	2969(PNG)
*Hermodice carunculata* (Pallas, 1776)	–	Alykes, Crete, Greece (35.4158°, 024.9875°), 5m	10.1	Anterior end	2 x 2 h in HMDS	air	0.3°	1200	155	0:32	8.922	877 (BMP)

To test the effect of different contrast-enhancement methods on the imaging results and tissue characteristics, several samples were treated with one of the following methods: a) tissue staining with 1% iodine in 96% ethanol; b) tissue staining with 0.3% phosphotungstic acid (PTA) in 70% ethanol; c) desiccation with Hexamethyldisilazane (HMDS). Protocols for both iodine and PTA staining follow Metscher (2009a). In both solutions, smaller samples were stained in 2 ml for 24 hours to several days, larger samples in PTA required longer staining (up to 3 weeks) in larger amounts (10 ml), the solution was renewed every five days to allow PTA to penetrate into the tissue. Samples treated with HMDS were left in the chemical for two to four hours, in the larger specimen (*Hermodice carunculata*) the chemical was renewed after two hours. The amount of HMDS and the treatment time depends on the size of the specimen: as a general guide, an amount twice the body volume of the specimen was used. Afterwards, specimens were removed from the chemical and left to dry for several hours, causing them to desiccate while retaining their morphology. Details on treatment for each specimen are presented in Table 1.

Wet samples were scanned in heat-sealed 200 µl polypropylene pipette tips, either in ethanol or in air. The top of the container was sealed with a plasticine cap to prevent the specimen from drying out during scanning (for a similar setup see Metscher 2009a). Samples dried with HMDS were partially enclosed in a small piece of styrofoam which in turn was mounted on a thin metallic sample holder. For assessing the quality of the scans with regard to distinguishing features, in this study only the anterior end of most worms was scanned. Scanning only the anterior end reduced scanning time and allowed us to choose a higher resolution. In polychaetes, the anterior end usually comprises most diagnostic characters, thus allowing us to assess the usefulness of the scans based on taxonomic criteria.

### Image acquisition

Samples were imaged with a SkyScan 1172 microtomograph (http://www.skyscan.be/products/1172.htm ) at the biodiversity laboratory of the Hellenic Centre for Marine Research. This system uses a tungsten source with energies ranging from 20–100kV and is equipped with an 11 megapixel CCD camera (4000×2672 pixel) with a maximal resolution of <0.8 µm/pixel. Specimens were scanned at a voltage of 60 kV with a flux of 167µA and scans were performed for a full rotation of 360°. Except for *Alitta succinea*, for which a camera pixel binning of 2 × 2 was chosen, images were always acquired at highest camera resolution. Individual scanning parameters can be found in Table 1. Projection images acquired during the scanning process were subsequently reconstructed into cross sections with SkyScan’s NRecon software which employs a modified Feldkamp’s back-projection algorithm. Sections were always reconstructed from the total number of projection images (360°) to obtain a greater level of detail, other reconstruction parameters were chosen individually for each sample. In case of strong density differences in the scanned sample, the upper limit of the grey scale histogram was lowered to unite very dense values. This causes dense values above the set limit to be assigned to the same grey scale value without differentiation and allows softer (less dense) tissues to be visualised with greater detail. The lower limit of the histogram was set at the value for the surrounding medium (air or ethanol). To reduce the size of the resulting images, only areas containing relevant data (regions of interest) were reconstructed, thus excluding the surrounding air or enclosure medium.

### Molecular analyses

*Hediste diversicolor* (O.F. Müller, 1776) specimens collected in Tsopeli lagoon in Amvrakikos Gulf (Western Greece) were sequenced before and after X-ray exposure in order to assess whether the radiation had an effect on the 16S rRNA sequence obtained. Samples were exposed either to high energy of radiation for a relatively short time (100kV for 1.5h) or repetitively exposed for three cycles of 12 hours at medium energy (12h, 24h, 36h at 60kV). In the latter series, some tissue was removed from the specimen for DNA extraction after each cycle. A fragment of the 16S rRNA gene (~ 500 bp) was amplified using a primer pair designed for polychaetes: 16SAN-F (TACCTTTTCATCATGG) and 16SEU-R (ACCTTTGCACGGTCAGGRTACCGC) (Zanol et al. 2010). Genomic DNA concentration of samples was calculated using a Nanodrop 1000 spectrophotometer (Wilmington DE, USA) on wavelength 260 and 280nm. Polymerase Chain Reactions (PCRs) were performed in a final volume of 20 µl containing 0.4 units KAPA Taq DNA polymerase (Kapa Biosystems, Inc, USA), 2 µl PCR buffer 10x, 4.5 mM MgCl2, 2 mM dNTPs, 10 pmol of each primer, and 0.5 µl (~5 ng/μl) of DNA template. Amplification was performed in a MJ Research PTC-200 Thermo Cycler (Harlow Scientific, USA) programmed as follows: initial denaturation cycle at 96°C (4 min) followed by 35 cycles of denaturation at 93°C (45 sec), annealing at 52°C (1 min) and extension at 72°C (1 min); last cycle was followed by a final extension at 72°C for 7 min. The sequences were processed with MEGA v. 5 software (Tamura et al. 2011). Obtained sequences were submitted to GenBank (Benson et al. 2005) under the accession numbers KC113440-KC113445.

### Processing, presentation and dissemination of image data

**Two-dimensional images:**

All resulting datasets of cross sections were post-processed with the CTAnalyzer (CTAn) software (SkyScan, Kontich, Belgium) by selecting a Region of Interest (ROI) containing the sample but removing further superfluous information, thus creating a dataset of reduced size. To obtain a three-dimensional representation of the sequence of cross section images, the data were visualised with two different volume rendering software packages: both CTVox (SkyScan, Kontich, Belgium) and the free software Drishti were employed (http://anusf.anu.edu.au/Vizlab/drishti/ ). Volume rendering displays the data by assigning a colour value and an opacity value to each data point (voxel) in the dataset. By changing these transfer values, different features of the dataset can be visualised and explored. Density-based false-colour renderings were applied to the data where this was considered helpful to visualise structures. Isosurface models (geometrical representations of surfaces of equal values) were created with Amira v. 5.2 (Visage Imaging, Berlin, Germany). Two-dimensional images were extracted as bitmap files with the image export function of the respective software and consequently cropped to final dimensions and minimally edited in Adobe Photoshop to enhance contrast (adjusting image levels and curves) or transform colour tint (adjusting hue and saturation), as well as to add annotations.

**Interactive volumetric data:**

The *Lumbrineris latreilli* dataset was first processed with custom functions of CTAn (thresholding, smoothing, noise removal) to isolate the jaws from the surrounding tissue and saved as a separate dataset. This new dataset was subsequently loaded into the free image editor Fiji (http://fiji.sc ) and reduced in size to a stack of 320 images with dimensions of 205 × 173 pixels. These bitmaps were converted into TGA (Truevision Graphics Adapter) files with the free ImageMagick tool http://www.imagemagick.org ) and rendered with the C++ programmevolren (Ruthensteiner et al. 2010) which is based on the plotting library S2PLOT (Barnes et al. 2006). With this library, the data were converted into a three-dimensional VRML (Virtual Reality Modelling Language) object, accompanied by a PNG (Portable Network Graphics) file of each angular view. A corresponding script provided by the authors ensures that the correct view is rendered when the object is manipulated. In the volren script parameters were adjusted to AMIN=0.0001 and AMAX=0.1 and the colour map “iron” was chosen.In the resulting VRML file the texture transparency parameter was changed from 0.4 to 0.1 throughout the file in order to increase the contrast of the embedded model in the PDF (Portable Document Format) file.

**Interactive surface description models:**

Using the segmentation editor of Amira, features of interest were manually segmented (“labelled”) with the brush tool. For each feature a new LabelField was created, thus allowing the different objects to be manipulated separately at later stages. Labelled features were converted into surfaces with the SurfaceGen module and where the number of polygons was too high (>1,000,000) they were reduced with the Simplifier tool to increase computation efficiency during further processing. Amira’s SmoothSurface module did not produce satisfactory results in models with small detailed structures, since the module does not allow for selective smoothing and small structures disappeared after the application of the module. The models were, therefore, exported as OBJ (Wavefront Object) files and further processed with Blender 2.63a (http://www.blender.org ), a high end, open source, 3D design program. In Blender, the surface was cleaned of artefacts by applying the Vertices’ Relaxation and the Vertices Smoothing operations, which replaced the original model’s points (vertices) in average positions between them, thus smoothing surface anomalies. This process was repeated where necessary, until the model’s surface appeared smooth and even, without alienating main parts or the overall morphology of the model. If surface noise still persisted in parts of the model, then a second, manual part of cleaning was applied. In this case, specific parts of the model were selected individually and corrected by using additional tools in Blender (e.g. sculpting smooth brush). Some geometrically elegant (small, narrow, light) parts of the model (e.g. chaetae) could not be cleaned or were destroyed by the above techniques, in this case these parts and elements were reconstructed (remodelled) manually and added again to the model.

**Videos:**

The videos of 3D volume renderings were created with CTVox, using the flight recorder function, and saved as an AVI (Audio Video Interface) file. The video of cross sections through the sample was created by loading the image stack of the sections into Fiji and re-sampling the data to reduce image size. The resulting new stack was exported as an AVI video file with 20 frames per second. To be able to embed the videos into the PDF document, the AVI files were converted with an online conversion software (http://www.online-convert.com ) into FLV (Flash Video) files with a bit rate of 1000kb/s and a width of 400px.

**Embedding multimedia and interactive objects into PDFs:**

Both surface models and volume renderings were embedded into the PDF with the Acrobat X Pro 3D PDF Converter Suite (Tetra 4D, Seattle, USA). The 3D Reviewer module was used to define colours and views and to add annotations. The resulting data were again exported to PDF format, specifying in the export options “PCR tessellation” and “Compress tessellation”. These options reduced the final file size to about one third of the original object size. The proper rendering of the volumetric data required also the inclusion of a JavaScript file which is distributed along with the S2PLOT library (http://astronomy.swin.edu.au/s2plot/peripheral/s2plot.js ). Videos were added with Adobe’s Add Multimedia function. The process of embedding models and multimedia content into PDFs is relatively straightforward, detailed descriptions are provided by Barnes et al. (2006), Ruthensteiner and Hess (2008), Kumar et al. (2010) and Ruthensteiner et al. (2010).

**Electronic publication and data dissemination:**

All media included in this publication as well as supporting material (surface models, image files, videos) are published under a Creative Commons Attribution 3.0 (CC-BY) licence in a Virtual Research Environment, the Polychaete Scratchpad (http://polychaetes.marbigen.org ). The full volumetric datasets have been archived at the Dryad Data Repository (http://datadryad.org , doi: 10.5061/dryad.84m54). Since the direct inclusion of interactive, three-dimensional models in web pages is still in its infancy and requires specific browser and driver configurations on the client side, interactive models have been included as separate PDF files on the web site of the journal as well as on the Scratchpad site, thus allowing the majority of users to access this content. Most Acrobat products (Reader, Professional) from Version 8 onwards support the display of embedded media. However, specific versions of the software still show incompatibility problems and some users might therefore encounter problems viewing the interactive content. In this case, it is recommended to download the multimedia content (videos, interactive models) from Dryad, or the Polychaete Scratchpad, and view it with other software (e.g. a multimedia player).

## Results

### Information content of the datasets

***Lumbrineris latreilli*:**

**Figure 1.**
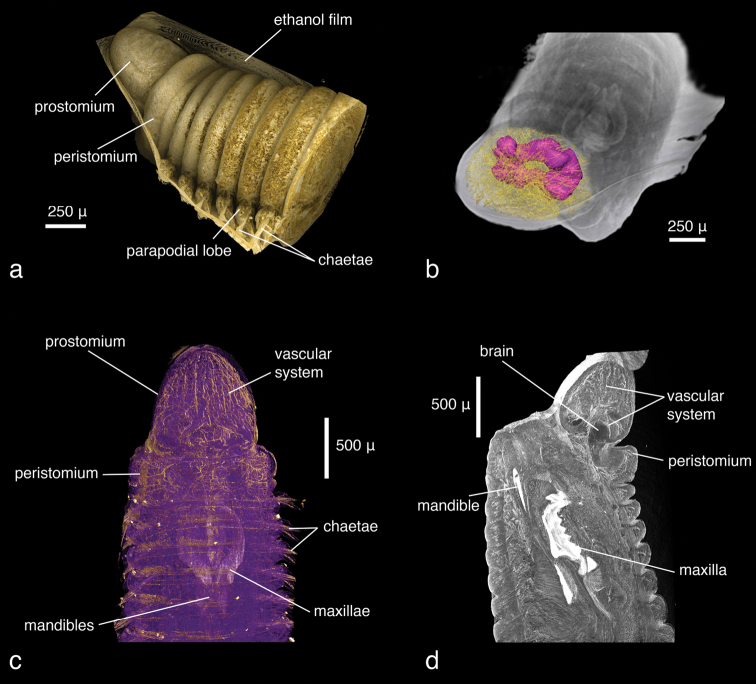
*Lumbrineris latreilli*, **a** false-colour volume rendering of the anterior region, dorso-lateral view, specimen partly covered by a thin film of ethanol **b** surface model of the vascular system (yellow) and brain (purple) superimposed on volume rendering of the worm, dorso-anterior view **c** false-colour, semi-transparent volume rendering of the anterior region, dorsal view **d** volume rendering of virtually dissected anterior region, lateral view.

**Figure 4.**
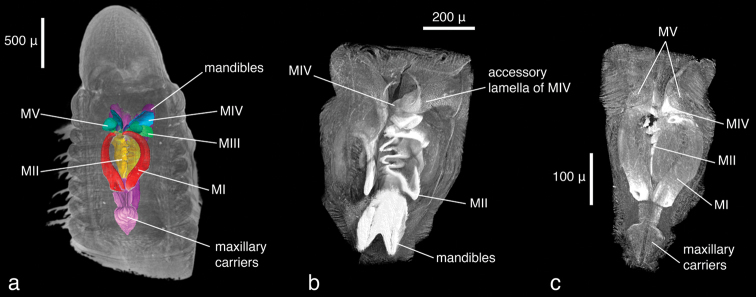
*Lumbrineris latreilli*, volume rendering of **a** anterior regionwith maxillary apparatus superimposed as a surface model, dorsal view **b** virtually dissected jaw apparatus, ventral view with mandibles partly removed **c** same, dorsal view.

Only the anterior end of the specimen was scanned, the total length of the visualised part being 2.4 mm. At a resolution of 1.9 µm/pixel, the smallest discernible structures are about 4 µm in size. Since the specimen has been scanned in air without prior desiccation, a thin film of ethanol partially covers the body and obscures parts of the external morphology; however, general external characters (size and shape of larger features such as segments, head, mouth opening) are clearly recognisable (Fig. 1a). The shape of the parapodia is visible but obscured by the ethanol film. The number and arrangement of the chaetae can be discerned but the chosen resolution prevents finer details such as chaetal articulation or dentation from being captured. The internal anatomy, on the contrary, has been recorded in substantial detail. The resolution is high enough to allow the observation of even the fine structure of the vascular system in the anterior part of the head (Figs 1b–c). Likewise, muscular groups and even their fibres are clearly visible (Fig. 1d). Nervous tissues (brain, ganglia, nerves) have a very low density and are difficult to depict in detail in the volume renderings. However, large nervous structures can be visualised by applying appropriate transfer functions (Video 1) and through careful remodelling (Fig. 1b). The most prominent visible feature is the jaw apparatus, a calcified complex system of maxillae and mandibles (Figs 2–4). However, the maxillary pair MV, the maxillary carriers, as well as the accessory lamellae of mandibles III and IV appear to have a similar density to the surrounding muscle tissue. This makes it impossible to visualise them in the low-resolution interactive volume model included in this publication (Fig. 3). In the high-resolution dataset, however, they can be visualised by applying suitable transfer functions (Figs 4b–c), which allows them to be included in the surface model (Fig. 2). The accessory lamellae of MI and the connecting plates of MI and MII, described as “weakly sclerotised in *Lumbrineris*” by Carrera-Parra (2006b), cannot not be clearly discerned in the data.

**Video 1.** *Lumbrineris latreilli*, false-colour volume rendering, virtual dissection of anterior end, dorsal view. Video available for download in full resolution from http://polychaetes.marbigen.org/lumbrineris-latreilli-micro-ct-video .

**Figure 2.** *Lumbrineris latreilli*, surface model of the jaw apparatus. Terminology follows Carrera-Parra (2006). If viewed with Adobe Acrobat Reader (version 8 or higher), the interactive 3D-mode can be activated by clicking on the image, allowing the user to rotate, move and magnify the model, to isolate elements and to change the light settings.

**Figure 3.** *Lumbrineris latreilli*, volume model of the jaw apparatus. Note the absence of MV as well as the accessory lamellae of MI, MIII and MIV which cannot be displayed in this low-resolution version. If viewed with Adobe Acrobat Reader (version 8 or higher), the interactive 3D-mode can be activated by clicking on the image, allowing the user to rotate, move and magnify the model.

***Eunice* sp. (juveniles):**

**Figure 5.**
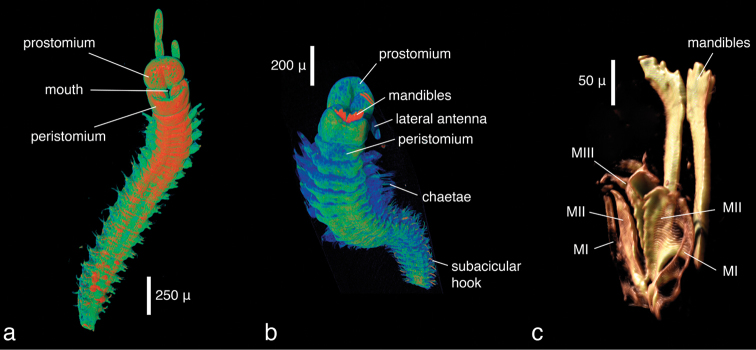
*Eunice* sp., false-colour volume renderings (colours indicate relative densities: blue: low, green: medium, red: high) of **a** iodine-stained specimen, ventral view **b** PTA-stained specimen, ventral view **c** false-colour volume rendering of virtually dissected maxillary apparatus (iodine-stained individual), dorso-lateral view.

**Figure 6.**
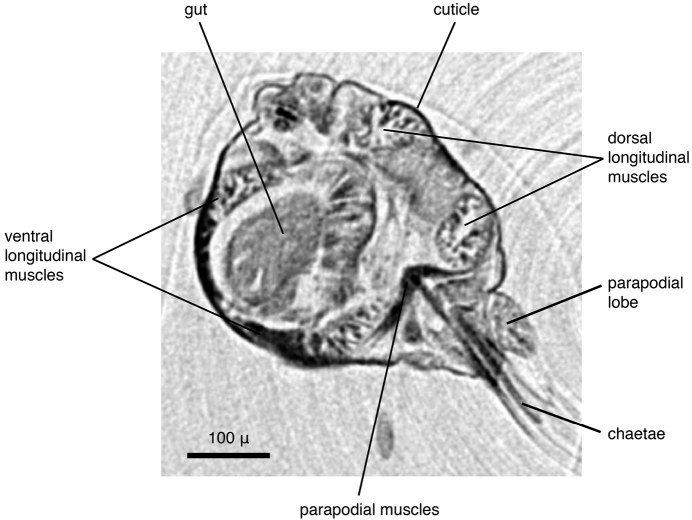
*Eunice* sp., transaxial cross section at mid-body (iodine-stained individual).

The specimens were similar in size and developmental stage, making them ideal for testing the effect of different tissue stains (discussed below). As in the *Lumbrineris* dataset, larger external features such as body shape, antennae and parapodia are excellently visible (Figs 5a–b), although the animals are smaller and the resolution is slightly coarser (ca 2.25 µm/pixel). No details of chaetae are visible, but the subacicular hooks are clearly depicted especially in the iodine-stained specimen (Fig. 5b) and the chaetal bundle is well visible in cross sections (Fig. 6). Internally, large organs and muscle groups are fairly distinct (Fig. 6), but the small size of the animal (width ca 0.3 mm) does not allow details that would be visible in histological sections to be discerned (e.g. vascular system, ganglia), with the exception of muscle fibres. The general structure of the jaw apparatus is evident, but finer details are difficult to see, and only MI–MIII can be unambiguously identified (Fig. 5c).

***Eunice roussaei*:**

**Figure 7.**
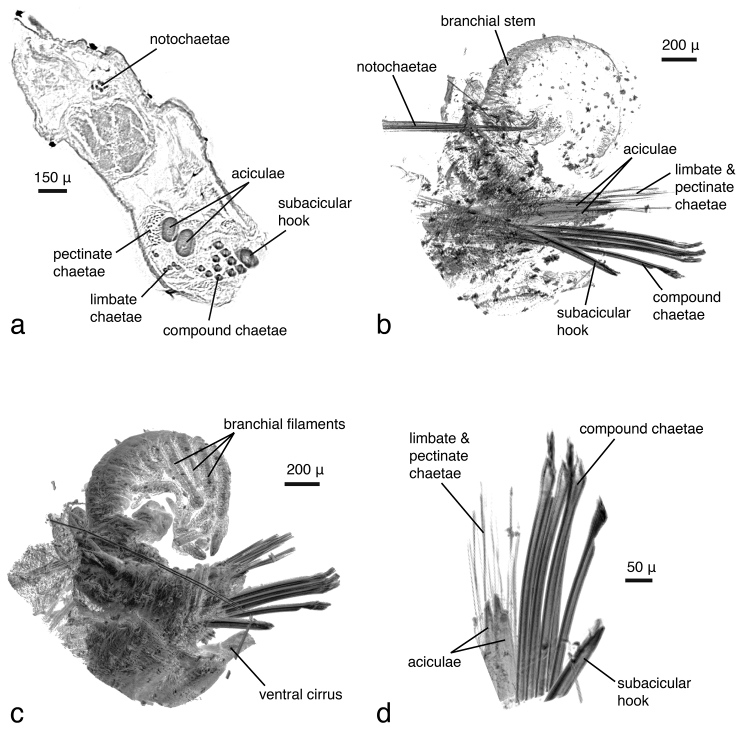
*Eunice roussaei*, mid-body parapodium, **a** cross section through parapodial base **b** semi-transparent volume rendering **c** opaque volume rendering **d** volume rendering of chaetae.

The dissected parapodium shows, especially in the cross sections, the exact arrangement of the different chaetal types (aciculae, subacicular hook, compound chaetae, supra-acicular limbate and pectinate chaetae as well as internal notopodial chaetae) (Figs 7a–b). External features, such as dorsal and ventral cirri, parapodial lobes, branchial stem and branchial filaments, can likewise be observed (Fig. 7c). Due to the position in which the specimen dried, the branchial stem is slightly recoiled, making the exact count of branchial filaments not straightforward; they are better delineated in the interactive, three-dimensional model of the specimen (Fig. 8). Details of chaetae (e.g. serration, dentation) are not visible (Fig. 7d). This is primarily caused by the coarse resolution of the scan (ca 2.9 μm/pixel), which prevents capture of these tiny details. Furthermore, because the specimen was dried and scanned in air, the chaetae vibrate slightly during each rotation step, resulting in a slight blur in the final images (Fig. 7d). Several internal structures are visible, such as muscle groups or connective tissues, but no blood vessels (e.g. in the branchiae) can be seen. Generally, most internal structures are difficult to identify, since the contrast between neighbouring tissues was equalised by the desiccation process.

**Figure 8.** *Eunice roussaei*, surface model of mid-body parapodium. Limbate chaetae, pectinate chaetae and notochaetae are not shown. Compound chaetae have been included as simplified, remodelled version and do not depict true shapes. If viewed with Adobe Acrobat Reader (version 8 or higher), the interactive 3D-mode can be activated by clicking on the image, allowing the user to rotate, move and magnify the model, to isolate elements or to change the light settings.

***Alitta succinea*:**

**Figure 9.**
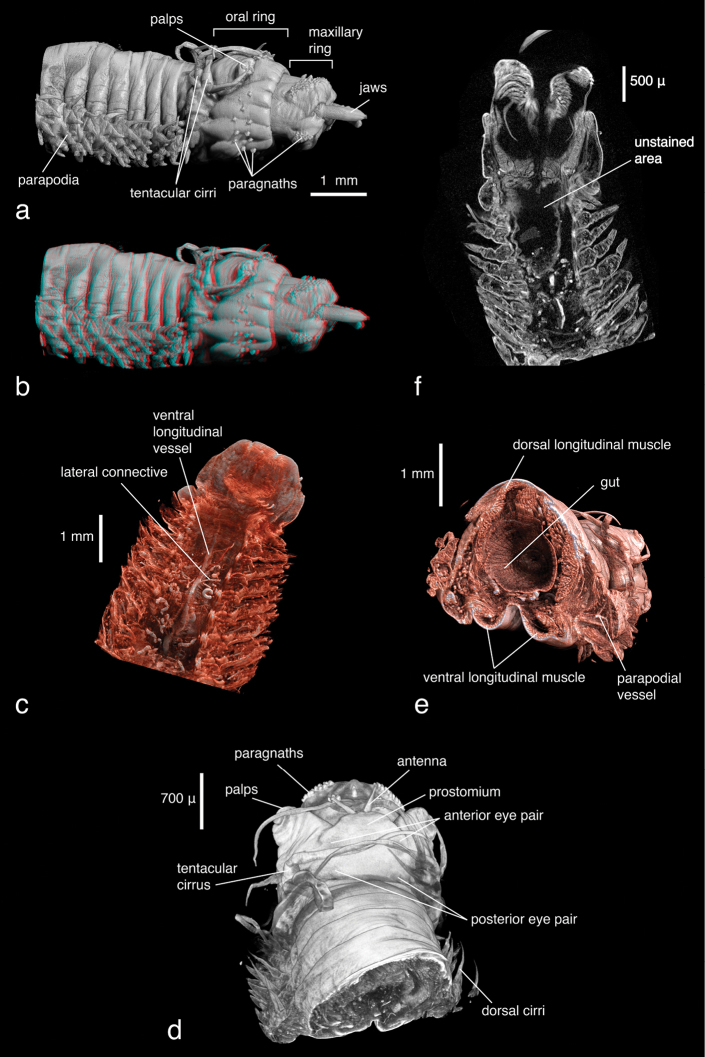
*Alitta succinea*, **a** normal view and **b** stereo view of volume-rendering of anterior region, lateral view (3D effect is revealed when viewed with red-cyan glasses) **c** coronal cross section of anterior region depicting areas of insufficient tissue staining with PTA. Black areas in pharyngeal region remained unstained and information is lost **d** dorsal view and **e** posterior-lateral view of false-colour volume rendering of virtually dissected anterior region **f** volume rendering of anterior region, dorsal view.

The overall morphology of the specimen, especially cephalic features (shape, appendages) and parapodia, can be clearly observed. The true three-dimensional structure of the parapodia, which consist of a complex arrangement of parapodial lobes, as well as dorsal and ventral cirri, are best distinguished in stereo display (e.g. with anaglyph 3D glasses) (Figs 9a–b, visible with red-cyan glasses). Chaetal structures cannot be observed, specimen preparation as well as scanning parameters were inappropriate for their proper visualisation. The pharynx in this specimen is everted, thus the determination of the shape of the jaws as well as of the paragnath shapes (conical) and distribution patterns—important taxonomic characters—is straightforward without having to virtually dissect the specimen (Fig. 9a). Since X-ray imaging is attenuation-based and thus records differences in density (or, depending on the energy, differences in the atomic number of the material), the colour of the paragnaths (another diagnostic character) cannot be observed. Eyes are visible as slightly darker spots on the epidermis, their density is apparently slightly different from the surrounding tissue (Fig. 9d). The internal anatomy is, as in the other samples, well visible. Major muscle groups can be discerned (Fig. 9c, e), as well as the vascular system. The latter seems to be discontinuous in parts (Fig. 9c), possibly a result of fixation in ethanol. The brain or other nervous tissues cannot be discerned. Apparently hollow regions in the pharyngeal area result from insufficient staining (Fig. 9f). The density of these unstained regions is too low to be visualised and the corresponding information is lost.

***Phyllodoce lineata* and *Phyllodoce* sp.:**

**Figure 10.**
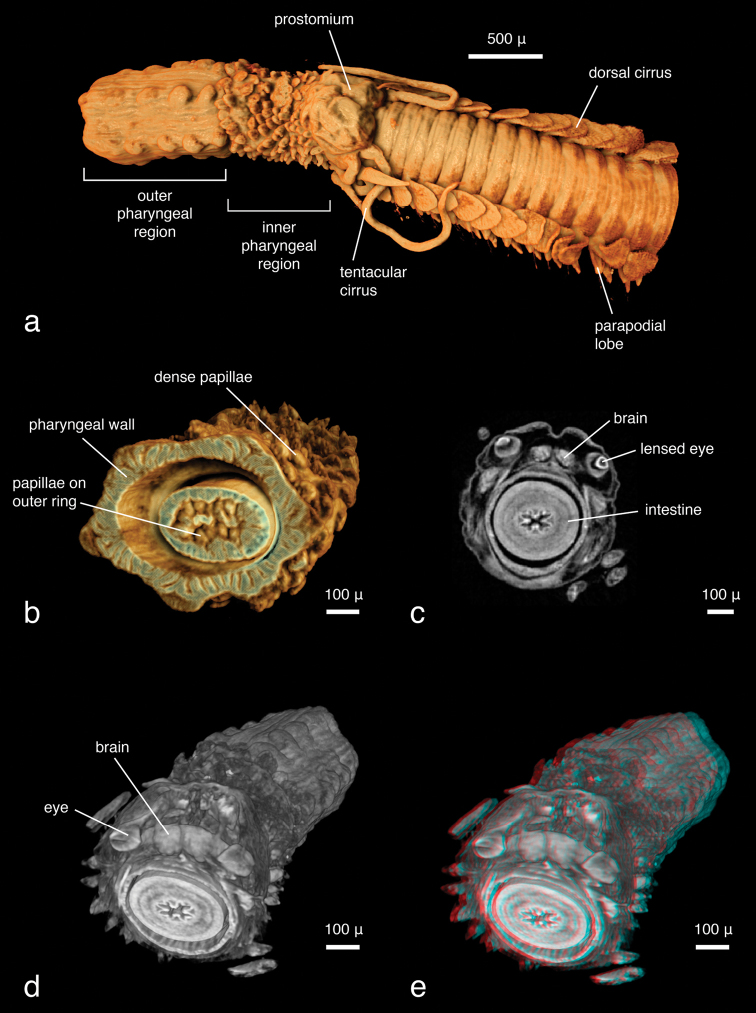
*Phyllodoce lineata*, **a** false-colour volume rendering of anterior region, dorso-lateral view**b** false-colour volume rendering of virtually dissected distal end of partly everted pharynx, focusing on terminal pharyngeal papillae, anterior view **c** transaxial cross section at eye level **d** volume rendering of virtually dissected anterior region showing the brain, dorso-posterior view **e** same, in stereo view (3D effect is revealed when viewed with red-cyan glasses).

**Figure 11.**
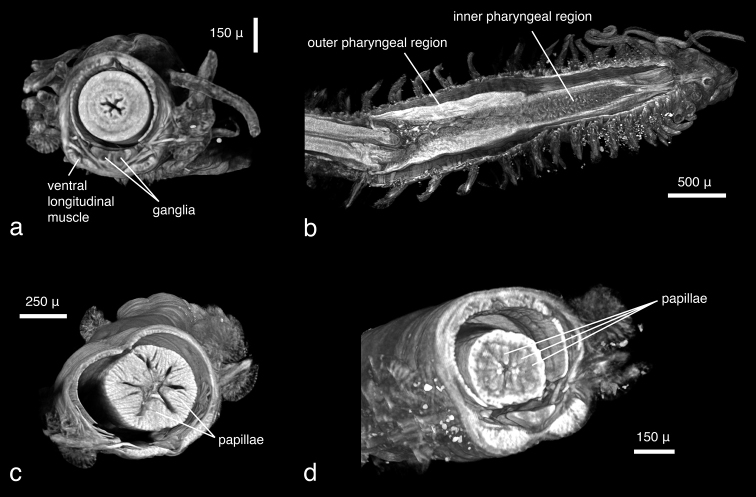
**a** *Phyllodoce lineata*, volume rendering of **a** anterior end, virtually dissected behind prostomial region, posterior view **b** *Phyllodoce* sp., volume rendering of virtually dissected pharynx, dorsal view **c** *Phyllodoce* sp., virtual dissection of distal pharyngeal subdivision with large papillae, anterior view **d** *Phyllodoce* sp., virtual dissection of proximal pharyngeal subdivision with small papillae, antero-lateral view.

*Phyllodoce lineata* can be identified to species level almost solely on the basis of the virtual specimen; all taxonomic key characters are visible, with the exception of mid-body parapodia which were not included in the imaged part of the specimen. However, the number, shape, length and arrangement of tentacular cirri and antennae, the shape (fused, partly fused, covered) of the prostomium and the anterior segments of the parapodia with dorsal and ventral cirri, and the position of chaetae can be observed (Fig. 10a), but finer details of the parapodial lobes and the chaetae cannot. The pharynx is partly everted, which makes the assessment of the arrangement of the pharyngeal papillae straightforward. The subdivision of the pharynx is well visible, with the proximal end being covered with scattered smaller papillae, the distal end with six rows of large papillae (Fig. 10a). The papillae around the pharyngeal opening can only be seen when the animal is virtually dissected (Fig. 10b). The eyes, containing lenses as in most phyllodocid species (Rouse and Pleijel 2001), appear as dense structures (Figs 10c–e, Video 2). Internally, all muscular features, the gastrointestinal tract, ganglia and the brain and large connecting nerves are well visible (Figs 10d–e, 11a, Video 2), especially when viewed in stereo display with red-cyan glasses (Fig. 10e). The other *Phyllodoce* specimen was imaged with a similar resolution and can thus be compared to the scan of *Phyllodoce lineata*. Morphologically, no differences between the two specimens are evident, however, the pharynx in *Phyllodoce* sp. is not everted. Virtual dissections reveal two subdivisions of the pharynx (Fig. 11b), a distal part covered with large papillae and a proximal part with small papillae. The cross sections of the pharyngeal regions show that the distal part has six rows of large papillae (Fig. 11c), however, neither the number nor arrangement of the smaller papillae can be determined (Figs 11d), making an unambiguous identification of the species impossible. Naturally, no colour patterns — a species-specific character in several phyllodocid taxa (Nygren et al. 2010, Nygren and Pleijel 2011) — are visible in any of the scans.

**Video 2.** *Phyllodoce lineata*, false-colour volume rendering, virtual dissection. Video available for download in full resolution from http://polychaetes.marbigen.org/phyllodoce-lineata-micro-ct-video .

***Syllis gracilis*:**

**Figure 12.**
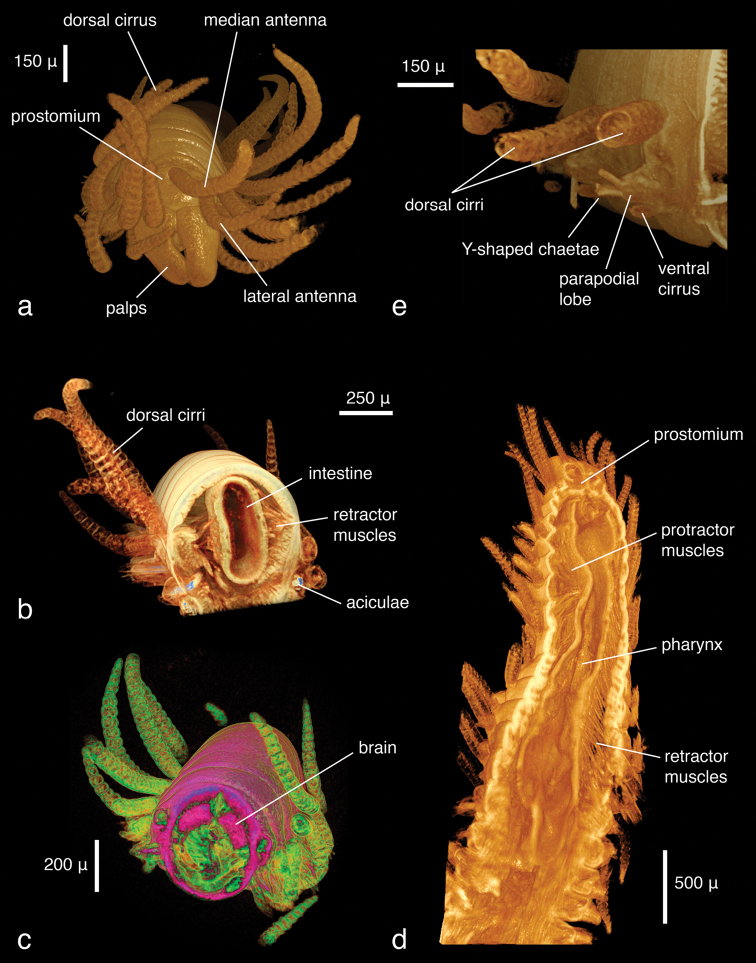
*Syllis gracilis*, false-colour volume rendering of **a** anterior region, anterior view **b** anterior region virtually dissected at pharyngeal level, posterior view **c** virtually dissected anterior region showing the brain, anterior view **d** virtually dissected region in front of proventricle, dorsal view; e) mid-body parapodia, posterior-lateral view.

**Figure 13.**
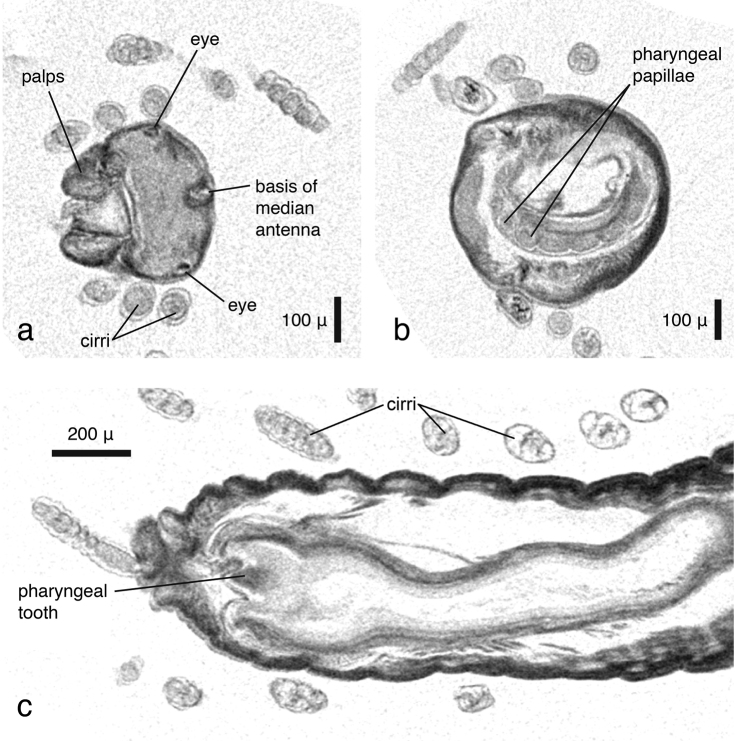
*Syllis gracilis*, cross sections, **a** coronal view showing prostomium, palps and posterior eye pair **b** transaxial view at level of pharyngeal opening, showing papillae around pharyngeal opening **c** dorsal view, pharyngeal opening and pharyngeal tooth. Double lines at borders of cirri are artefacts resulting from either movement of specimen during the scan or from settings during dataset reconstruction.

**Figure 14.**
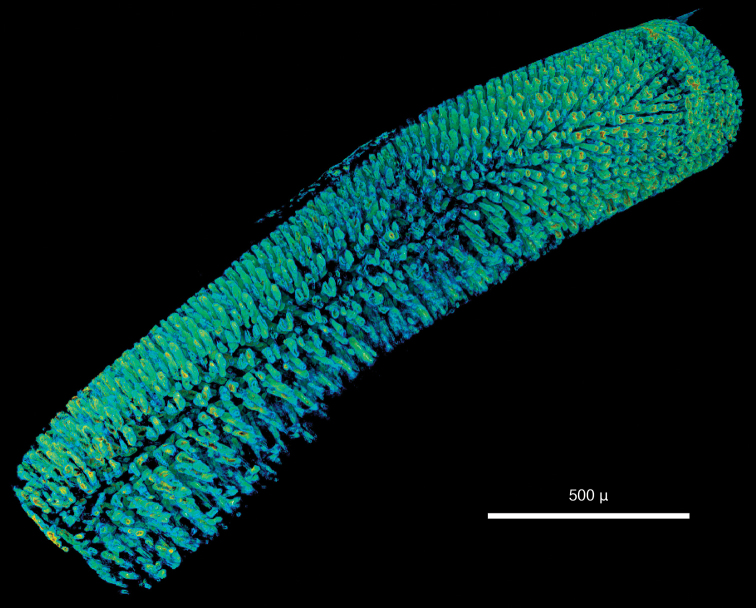
*Syllis gracilis*, false-colour volume rendering of virtually dissected proventricle.

As in the other data, large external morphological features of the specimen are well defined. Appendages, their articulation and number of articles are clearly depicted (Fig. 12a). Internally, features such as muscles (Figs 12b, d), the brain (Fig. 12c), the gastrointestinal tract (Fig. 12d) and muscle groups with their individual fibres can be identified. Other features such as eyes, pharyngeal papillae and the pharyngeal tooth are difficult to detect in volume renderings but can be observed in cross sections (Figs 13a–c). The length of the proventricle and the number of its muscle rows are likewise important diagnostic systematic characters in the family. The strong muscle fibres of the proventricle are extremely well visible, and through virtual dissection their three-dimensional arrangement as well as their number can be well observed (Fig. 14). However, one of the key characters in syllid systematics are fine differences in chaetal structures. These cannot be discerned with the present resolution; only the rather large and robust Y-shaped chaetae typical for *Syllis gracilis* are visible (Fig. 12e).

***Hermodice carunculata*:**

**Figure 15.**
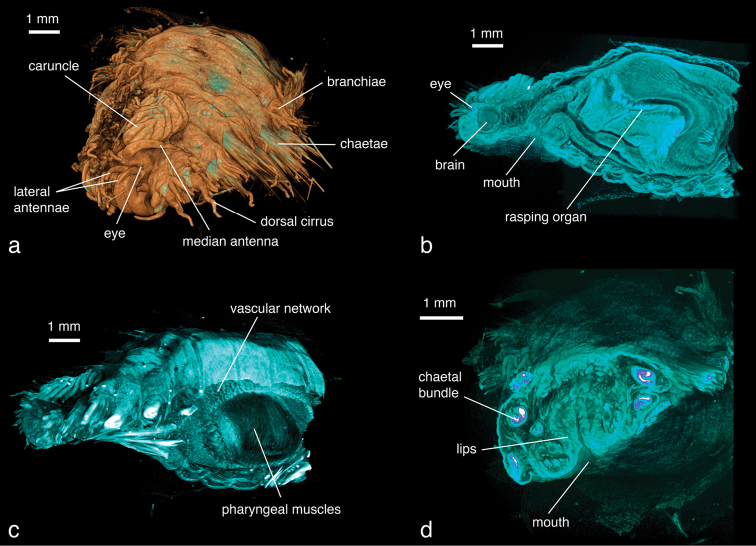
*Hermodice carunculata*, false-colour volume rendering of **a** anterior region, antero-dorsal view **b** virtually dissected anterior end showing pharyngeal structures, lateral view **c** virtually dissected anterior end, lateral view **d** virtually dissected anterior end at level of mouth opening, anterior view.

This specimen has been scanned at a low resolution (8.9 µm/pixel) because of its large size, thus details of smaller structures such as chaetae are lost. Individual chaetae can be differentiated, but at the base of the parapodia they appear as a thick, merged structure (Figs 15a, d). Larger external morphological features such as branchiae, parapodial structures, antennae and the caruncle are all clearly visible (Fig. 15a). Eyes, located under the cuticle, are visible in virtual dissection (Fig. 15b). Internally, the heavily vascularised area around the pharynx is well defined (Fig. 15c, Video 3). The complex folds of the pharyngeal system can be observed (Fig. 15b, Videos 3–4), as well as various parts of the muscular system (pharyngeal muscles, longitudinal muscles, parapodial muscles). The brain is clearly visible (Fig. 15b) and large nervous fibres such as the circumesophageal connective can be traced from the brain to the ventral ganglia (Fig. 15d).

**Video 3.** *Hermodice carunculata*, false-colour volume rendering, virtual dissection of anterior end, dorsal view. Video available for download in full resolution from http://polychaetes.marbigen.org/hermodice-carunculata-micro-ct-video .

### Contrast enhancement

Two different contrast enhancement techniques were employed in this study. These involved the removal of the surrounding liquid medium and tissue staining with electron-dense substances. Both result in an increased density difference between the specimen and the surrounding medium and thus produce sharp, contrasting images. However, different methods accentuate different morphological and anatomical features and thus the information content of the data differs accordingly. *Lumbrineris latreilli* was simply scanned in a sealed tube to prevent the specimen from drying out and X-rays were absorbed by the tissues according to their natural density differences and atomic number. Mineralised structures (jaw apparatus, chaetae) showed up most clearly, as well as the vascular system in the cephalic part of the animal. Muscle tissues are less dense but clearly visible, whereas nervous tissues (brain, ganglia) have almost no X-ray attenuation. Through careful observation, almost all anatomical features can be observed in the data (Video 1); however, external features are partly obscured by a thin ethanol film clinging to the specimen during scanning. By drying the specimen with HMDS, such artefacts can be avoided, since the specimen is fully desiccated while retaining its morphology. (e.g. Figs 7, 15). The process removes the liquid medium both from the cells and the surrounding area, resulting in a sharp overall contrast of all tissues. However, density variations between different tissues are less pronounced than in the wet specimen, creating difficulties in distinguishing neighbouring organs (Figs 10a, 15c–d). Scanning specimens in air can create other artefacts caused by slight vibration of protruding structures during rotation, such as the chaetae in the parapodium of *Eunice roussaei* (Fig. 7d). These artefacts become more pronounced with increasing magnification, since the effect of the movement becomes stronger.

Iodine and PTA bind to tissues and thus increase their X-ray energy absorption rate. The two stains generally bind to all tissues but exhibited different affinities to certain tissue types, staining them more intensely. Iodine seems to stain calcified structures and polysaccharides more strongly, whereas phosphotungstic acid is known to bind to certain proteins (fibrin, collagen) (Quintarelli et al. 1973) and stains the cuticle and muscle tissues more intensely. In the *Eunice* specimen stained with iodine, the cuticle (containing polysaccharides), the jaw apparatus (mineralised with aragonite) and the subacicular hooks were stained strongly compared to other tissues (Fig. 7b). The chemical composition of the subacicular hooks is unknown, but calcified chaetae are known to occur in other polychaetes (e.g. Amphinomidae (Westheide 1997), Pogonophora (George and Southward 1973)), thus their strong staining with iodine could indeed be caused by calcium components. In *Syllis gracilis*, the proventricle showed an increased density after staining, here the muscle fibres likewise contain calcium (Briggs et al. 1985) (Fig. 14). In the *Eunice* specimen stained with PTA, the cuticle, which consists of collagen fibres, was stained very intensely, especially in the anterior region of the animal (Fig. 7a). Muscular fibres surrounding the jaw apparatus, the longitudinal muscles and the parapodial muscles are likewise more pronounced with PTA than with iodine (not shown). Contrary to the iodine-stained specimen, the jaw apparatus and the subacicular hooks of the PTA-stained specimen were not stained and remain almost invisible (Fig. 7a). In the two phyllodocid specimens, PTA worked exceptionally well on the pharyngeal apparatus (Figs 10–11). In larger specimens, such as in *Alitta succinea*, the stain does not easily penetrate into the tissue—black areas in the images indicate unstained tissue (Fig. 9f). PTA penetrates slowly and tissue can bind large volumes of the chemical, so renewal every few days (or larger amounts) of the solution and longer staining times are required for larger specimens.

### Visualisation of results

Different approaches to communicate three-dimensional data through a scientific publication have been explored in this study. Firstly, two-dimensional images (screenshots) have been created from the volume rendering software, both normal images and stereo view images which, when viewed with red-cyan glasses, create a 3D-effect. Secondly, videos of interaction with the three-dimensional data have been created and directly embedded into the PDF version of this article. Thirdly, three-dimensional models have been embedded into the PDF which, when viewed with an Adobe Acrobat product, allow the user to interact with them (e.g. rotating, zooming). Both images and videos communicate predefined views of the data. Videos, however, contain a substantially larger amount of information than a single static image. The video of the sequence of cross sections through the data (Video 4) allows the user to investigate the full dataset in a very compact version, thus information is conveyed which would be impossible to include in a publication if only images were used. In other cases, videos allow the viewer to better perceive the spatial relation of structures to each other and understand their relative position and perhaps their functioning in the organism (Videos 1–3). The interactive models give the reader the greatest freedom to explore the data. Surface models provide an excellent method to present selected information on specific structures (e.g. jaw apparatus, parapodium with chaetae (Figs 2, 8)). The resulting models can be rotated, magnified and individual parts can be isolated, allowing the user to explore shapes and spatial relationships from all angles. However, surface models are more suitable for compact structures such as internal organs (see e.g. Ruthensteiner and Hess 2008) than for the display of fine details. In models with complex shapes and details the number of vertices increased dramatically, leading to very large file sizes which were not suitable for further processing. The relatively complex shape of the parapodium with branchiae is already at the limit of what a current standard desktop computer can process. Smoothing the surface and reduction of vertices reduced the data significantly, but automated smoothing or reduction risks eliminating small but taxonomically important details. The limbate and pectinate chaetae had to be omitted from the model since their thin structures were reduced to random dots along their length as soon as the number of vertices was reduced. The compound chaetae had to be completely remodelled and were finally included as simplified shapes, showing general shape and position but no details of structure. Manual post-processing of the model produced better accounts but requires detailed original data and a good knowledge of the morphology of the specimen. The process can furthermore become very time-consuming and the effort/outcome ratio has to be carefully considered. In the data produced during this study a hybrid approach was used: for most structures manual processing was considered not necessary, some characters were re-modelled as simplified shapes, others omitted. The optimal balance between model size and conveyed information will always depend on the research question being addressed. Software approaches, such as SPIERS (Sutton et al. 2012) that optimise the rendering of models with a high number of vertices can be an excellent solution if simplified shapes should be presented to the user.

**Video 4.** *Hermodice carunculata*, sequence of transaxial cross sections from post-pharyngeal chaetigers to the prostomium. Video available for download in full resolution from http://polychaetes.marbigen.org/hermodice-carunculata-slices-micro-ct-video .

To include the interactive volume data, the dataset had to be substantially reduced in size, resulting in a loss of many details. The embedded data are not a true volume rendering (based on a stack of images) but a pseudo-volume rendering: the software exports images from each angle of the volume-rendered objects and presents these to the user, creating the illusion of a three-dimensional object. The transfer function (and thus the information content) are predefined during model creation and cannot be changed by the end-user. The resulting model has thus not only limitations concerning the available detail of data but also towards the options for the user to explore the data, rotating and zooming being the only options of interactivity. The available transfer functions are furthermore not as sophisticated as in a desktop software. The slight density differences between the muscle tissue of the jaw apparatus and certain structures of the maxillary apparatus (maxillary carriers, accessory lamellae, MV) could not be visualised with the S2PLOT library (Fig. 5). The information value of these embedded objects is therefore limited, and in the present data the surface model is actually able to convey more information than the volume rendering.

### Molecular analyses

No differences between the 16s rRNA sequences from samples before and after scanning could be detected; moreover, the 16s rRNA sequences of samples with increasing exposure time to X-ray radiation were also identical. GenBank accession numbers of sequences before scanning and after different radiation energy and exposure time are listed in Table 2.

**Table 2.** GenBank accession numbers of the sequences obtained from *Hediste diversicolor* specimens before and after scanning.

**Sample code**	**Scanning time and voltage**	**GenBank accession number**
NER015	none (control)	KC113440
NER015	1.5 h, 100 kV	KC113442
NER063	none (control)	KC113441
NER063	12 h, 60 kV	KC113443
NER063	24 h, 60 kV	KC113444
NER063	36 h, 60 kV	KC113445

## Discussion

### Is micro-computed tomography suitable for the creation of cybertypes?

**Accuracy and reliability of information:**

Three-dimensional data resulting from micro-CT contain a wealth of information for systematists and taxonomists. The examination of characters in their natural position within the organism allows researchers to assess their true shape but also to infer functionality from morphological structures or even discover new diagnostic characters (e.g. Ziegler et al. 2010, Zimmermann et al. 2011). The non-destructive character of the technology allows examining internal features (even of very dense materials) without the need for dissection, leaving the original material intact. At the same time, characters can be investigated truly in three dimensions, whereas dissected material often has to be manipulated or squeezed for its adequate observation under a light microscope, thus rearranging the position of characters. The digital data allow the accurate identification of characters in three dimensions and relate them to each other. If needed, parts of the data can be isolated and examined separately, allowing their free manipulation without other body parts obscuring them (e.g. Figs 4b–c). With appropriate software, measurements can be performed in three dimensions, paving the way for a greater accuracy in these analyses which cannot be obtained with conventional methods.

Despite these obvious advantages, there are also certain limitations of micro-CT. A crucial point for research on small-sized organisms is image resolution which currently lies—depending on the system—in the range of 0.8 to 100 µm/pixel (although nano-CT systems can reach 0.1 µm/pixel (Wang et al. 2008)). This is a far coarser resolution than other techniques such as scanning electron microscopy, histological sectioning, cLSM or even light microscopy can achieve. Depending on the size of the specimen and the taxonomically important characters of the taxon, this resolution can prove sufficient. In many taxa, however, fine details in the micrometre range are important characters to distinguish species. These structures might be at the resolution limit of current micro-CT desktop scanners and cannot be adequately displayed. In some cases, the proper observation of characters might also be restricted due to their positioning during the scan. Under a stereo microscope specimens can be twisted or stretched, and obscured features can be made visible by careful manipulation with forceps. The data resulting from X-ray scanning are of course static, since only one position of the animal is imaged in a scan. Virtual dissections or measurements can partly overcome the problems, but in some cases the desired feature might simply not be visible in the data. Another limitation of micro-computed tomography is its inability to detect true colours. Pigmentation patterns can contain valuable taxonomic information in many taxa, but with current systems this information is not available. Recent developments of hybrid systems combining micro-CT with photon counting (Qiong et al. 2012) show, however, the first promising results of true-colour micro-CT imaging and might become a standard component of desktop scanners in the future. Until such hybrid systems become widely available, a solution for creating true-colour 3D models would be to use photographs or laser scans of the organisms and wrap these around the surface models to create realistic surfaces. However, these options require a substantial amount of manual post-processing and familiarity with 3D creation software.

**Figure 16.**
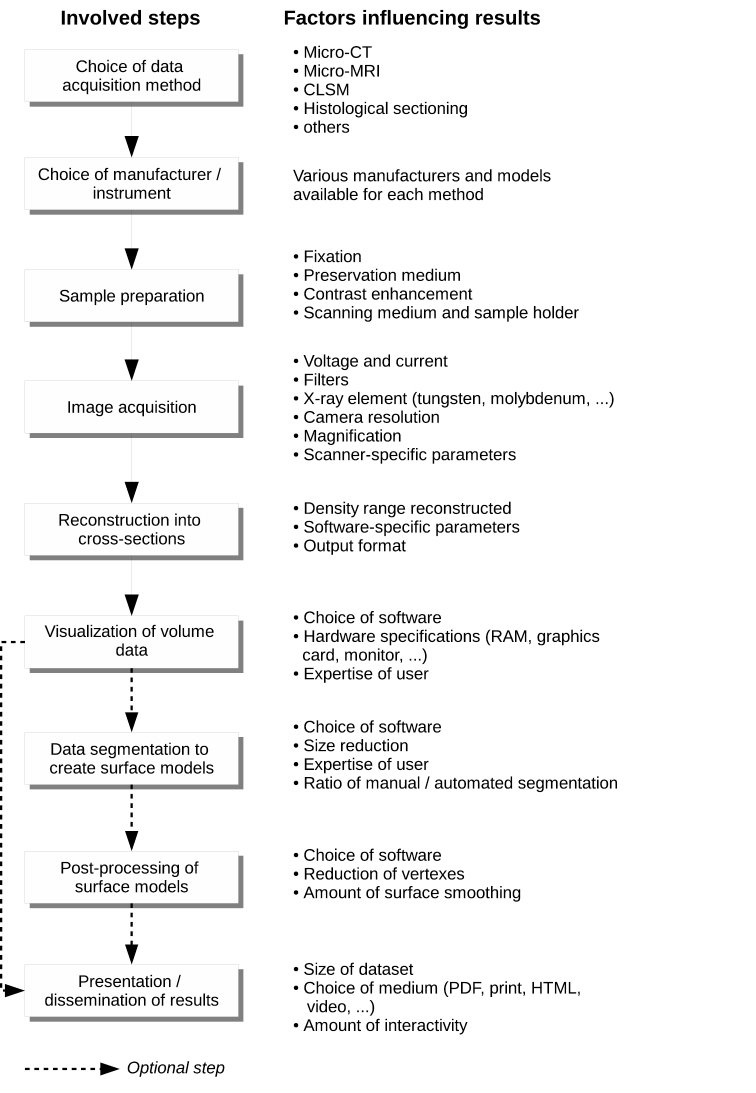
Diagram of the image acquisition process from the choice of method to the final presentation of the data, including factors influencing the outcome and information value of the results.

Apart from inherent limitations of the technique, the information value of a dataset also depends on a range of parameters and settings during the image production process. Besides artefacts which might be created during image acquisition (Abel et al. 2012), each step from the acquisition to the final presentation presents the user with a number of settings and choices, each of which can influence the final results (Fig. 16) and create problems of comparability between datasets. Since at each step a level of subjectivity is added, the closest approximate to raw data in this chain—the initial volumetric data—are most suitable to represent a virtual type, although the settings during sample preparation, scanning and reconstructing already determine much of the information content. By this definition, derivative data products, such as the interactive surface and volumetric models included in this publication would not qualify for a cybertype. However, the ultimate target which governs these settings and choices is to obtain the optimum result and an adequate view of the character(s) in question. In disciplines other than zoology, different conventions might be more adequate (e.g. Sutton et al. 2012).

**Effects on physical specimens:**

Micro-CT is commonly characterised as a non-destructive imaging technique, and indeed neither does the specimen need to be physically manipulated before scanning nor does the exposure to X-ray energies have any visible effect on the morphology of the specimen. However, nothing is known yet about possible tissue damage at the cellular level or after elongated or repeated exposure, so whether the technique is absolutely non-destructive remains to be proved. A certain risk of altering the specimen’s characters lies in their preparation for image acquisition, specifically in methods for contrast enhancement which might irreversibly change tissue characteristics. Although micro-CT does not *per se* require contrast enhancement, such treatment might be necessary, especially when scanning soft-bodied organisms. Tissue staining can lead to excellent results and sharp image contrast (Metscher 2009a, b) but no universally applicable protocols for the removal of these stains exist so far. The selectivity of stains towards different tissue types particularly enhances the contrast of certain tissues, whereas signals of other tissues are suppressed and show up less clearly or not at all in the scans. This alteration of natural tissue contrasts renders the specimen potentially useless for future micro-CT examinations with a different purpose. Tissue staining should therefore be used with care, especially if the data are intended for general purpose studies and/or if the material is valuable, since the long-term effects of these chemicals remain yet unknown. On the contrary, specimens scanned in air (either wet or dried) can simply be immersed again into the preservation medium after scanning and are thus available both for imaging methods and for investigation through traditional microscopy techniques, but again not all samples are suitable for this type of treatment. The absolute impact of each method is almost impossible to predict due to the large diversity of tissue types, chemical components and material combinations existing in invertebrates (Faulwetter et al. 2012). Protocols for best practice have, therefore, still to be identified experimentally for each species. The most suitable method is always determined by a number of factors such as the characteristics of the sample (density, size, shape), the surrounding medium and the scope of the study (Table 3). As far as indicated by the results of the current study, X-ray radiation induced by micro-CT seems not to have affected the molecular identity of the specimens, at least no effects of the radiation on the sequenced fragment of the 16S rRNA gene could be detected. Neither exposure to high energies nor repetitive exposure seems to have caused any mutations in this specific part of the molecular material. Previous studies attempting to determine whether exposure of preserved tissue to X-ray radiation causes a fragmentation of the DNA have reached contradicting conclusions (Götherstrom et al. 1995, Grieshaber et al. 2008, Paredes et al. 2012). As Paredes et al. (2012) point out, the findings of Götherstrom et al. (1995) and Grieshaber et al. (2008) might however be biased by the setup of the experiment. Götherstrom et al. (1995) examined the effect of X-ray radiation on the ability to amplify DNA fragments from pig bones, basing their conclusions on the results on the differences of PCR products brightness in an agarose gel. Results of Grieshaber et al. (2008) did not show any significant effect of the radiation on the DNA amplification with RT-PCR; nevertheless both studies conclude that exposure to radiation had caused degradation of the DNA. Paredes et al. (2012), by comparing pre- and post-CT DNA fragmentation profiles of preserved bird skin, did not detect significant differences of DNA quality before and after scanning, concluding that no quantifiable DNA fragmentation was induced by exposure of the sample to X-ray. However, all studies use different exposure times and energies as well as different methods to detect the effect of radiation on the genetic material, thus all these results are only indicative. Furthermore, none of the previous studies used sequencing data to compare the X-Ray effect on DNA samples. If the methodology is to be applied more broadly, further experiments are clearly needed. Likewise, the possible effect of staining substances on the molecular material should be tested; however, DNA of specimens subjected to different contrast-enhancement methods could be successfully extracted and amplified in previous experiments (Austin and Dillon 1997, Faulwetter et al. 2012). The above aspects of the potential impact of the micro-CT technology on preserved biological material must be entirely resolved before this method is suggested for wide use on the specimens deposited in museums. This holds especially true for type-material of nominal species which is unique by definition.

**Table 3.** Overview of different contrast enhancing techniques, their applications and limitations.

**Method**	**Use cases**	**Limitations**	**Reversibility**
Removal of preservation medium, scan in humid state	Natural contrast between different tissue types should be kept<br/> Specimen cannot be stained<br/> Specimen contains dense parts and only these should be visualised	Remaining drops of liquid between external features might obscure details<br/> Specimens might dry out if scanning time is too long<br/> External body parts such as branchiae, membranes, might collapse into unrecognisable shapes<br/> Soft tissue inside very dense structures cannot be visualised appropriately	Immerse into preservation medium
Drying (HMDS, possibly also critical point drying or freeze drying)	Specimen contains both dense parts and soft tissue and both should be visualised<br/> Specimen cannot be stained nor scanned in a humid state	Specimens become fragile, external body parts might break off<br/> Long appendages vibrate during rotation and create blur<br/> Natural contrast between tissues is reduced, individual organs might be difficult to separate	Immerse in >95% ethanol
Tissue stains (e.g. iodine, PTA, silver staining)	Specimen contains both dense parts and soft tissue and both should be visualised<br/> Specimen is very small or fragile<br/> Specimen cannot be removed from liquid medium<br/> Specimen has appendages that could vibrate in air or collapse to body when liquid medium is removed<br/> Only certain tissues should be visualised (selective staining)	Natural contrast between tissues is reduced, individual organs might be difficult to separate<br/> Tissues stain selectively, some tissues might not show up at all in the image<br/> PTA: large specimens need very long staining times and large amounts of stain<br/> Iodine: soft tissue does not stain well when specimen contains large calcareous structures (e.g. mollusc shells, serpulid tubes)	unknown

### Data access and curation of cybertypes

Three-dimensional data can be communicated in various ways and through various media. Their true potential lies, however, in the wealth of information that a full volumetric dataset offers to the skilled researcher. Volumetric datasets can easily reach a size of several gigabytes per dataset (Table 1), a fact that poses new challenges concerning the management, archival, backup and dissemination of these data. Currently, the community lacks infrastructures, standards and policies that allow the adequate curation of three-dimensional data (Rowe and Frank 2011). An urgent priority is the creation of an infrastructure for sharing these data and encouraging their reuse. Although a number of morphological databases exist at present (Ziegler et al. 2010), only few focus on the curation and visualisation of three-dimensional morphology data (e.g. Digimorph – http://www.digimorph.org ), Digital Fish Library – http://www.digitalfishlibrary.org , Berquist et al. 2012), and none acts as a broad-scale repository of high-resolution volumetric datasets. Apart from these archives, hundreds of three-dimensional datasets of biological specimens have already been produced but remain inaccessible to the research community (Ziegler et al. 2011a, Boistel et al. 2011). The development of standards and protocols for archiving and disseminating three-dimensional data as well as the creation of centralised registers to make the information retrievable remains an immediate priority (Ziegler et al. 2010, Rowe and Frank 2011) and is crucial for the future success of these developments. In this context, natural history museums and other large natural collections will have to play a central role, not only by digitising their collections and thus massively producing data (as already exemplified by recent efforts (Smith and Blagoderov 2012)), but also by taking the lead in the development of standards and software for virtual museum collections and curation of cybertypes (for a discussion on the lack of standard file formats for interchange see Sutton et al. 2012). This leads to another important issue—the current lack of standards to properly document and exchange volumetric data. Without metadata, datasets are neither retrievable nor interpretable. The medical community has developed the DICOM standard (Digital Imaging and Communications in Medicine, http://dicom.nema.org/ ) which contains both format definitions and a communication protocol for the description and exchange of volume data. However, this standard is not as universally implemented as one might expect, with different formats and versions being used by different parties (Mildenberger and Jensch 1999, Pianykh 2012). Furthermore, to what extent the DICOM specifications are in compliance with the purposes of the taxonomic community remains to be investigated, thus DICOM might or might not prove to be a suitable format for volumetric data definition and exchange. Another limiting factor for the optimal use of volumetric data is that powerful computing capabilities are required for the visualisation, exploration and analysis of large data sets. Although computing power is becoming increasingly more inexpensive, large-scale analyses and comparisons of datasets will likely be limited to high-performance computing centres. Virtual laboratories providing remote access to these facilities could however give a true boost to widespread usage of these data (Ziegler et al. 2010). System architectures to provide rapid access to three-dimensional data are already being developed by the informatics research community (e.g. Engel et al. 1999, Kaupp et al. 2002, Prohaska et al. 2004, Congote et al. 2009, 2012). Finally, a third challenge for the biomedical informatics community is the incorporation of three-dimensional information on web sites. A major bottleneck for the publication of volumetric data is still their large size which has to be transferred to the client for visualisation, but also the lack of native integration of this data into HTML standards. Developments such as the VAXML standard (Sutton et al. 2012) could potentially become the basis for such integrations. The near future will see several promising developments such as the Arivis WebView browser (http://webview3d.arivis.com/ ) or Voluminous, the web-based version of Drishti (under development, presentation at https://sites.google.com/site/ozvizworkshop/ozviz-2011 ).

### The future of virtual taxonomy

The increasing availability of accurate, three-dimensional virtual representations of biological specimens offers an exciting range of new research opportunities and will significantly accelerate access to first-hand morphological information, thus helping to overcome one of the major bottlenecks in systematic and taxonomic research: the continuous availability of type material to all potential users simultaneously. At present, although virtual specimens in most cases cannot—and should not—replace physical type material, often the desired information can be obtained from a virtual representation. In this study, none of the scanned specimens comply to the hypothetical requirement for a virtual type to provide as much information as the original material. However, different imaging methods (e.g. photography, nano-CT, MRI, OPT) can be employed to produce complementary datasets, and sophisticated future methods could provide a seamless integration of different datasets, incrementally loading additional data when zooming in or focusing on certain characteristics. The availability of information-rich cybertypes would not only protect the actual type material from loss or damage through careless handling, but would also provide simultaneous access by multiple users to the material. This approach would also provide a way to access collections where local restrictions prevent removing specimens from the institution or country.

The increased creation of three-dimensional taxonomic data will also inevitably influence the way taxonomic data is published. Embedding data as interactive, three-dimensional objects into publications will likely become a standard to usefully convey information. With the recent amendment of the International Code of Zoological Nomenclature (ICZN) that allows taxonomic treatments to be published exclusively electronically (International Commission on Zoological Nomenclature 2012), multimedia and interactive data can be embedded directly into the publication. This allows these large datasets to be embedded within the presentation of the paper as a single “coherent scientific report” (Ziegler et al. 2011b). With these regulations, an important milestone has been reached in the process of transforming taxonomy into a cyber-discipline. However, if the concept of virtual type material types is to be officially established, further changes to the ICZN will have to be made that regulate the use of cybertypes to complement physical type material. Specifically, Article 72, which regulates the type concept in nomenclature, should be amended to include the definition of the concept of a “cybertype”, “e-type” or “virtual type” and provide recommendations upon the nomenclatural status, electronic format, access and longevity of such datasets, as well as ensuring that they are made accessible to the public domain under a licence that ensures open access and encourages the creation of derivative works. Furthermore, as Wheeler et al. (2012) point out, regulations concerning registry of cybertypes in central access points such as ZooBank should be included. Such regulations will allow entirely new ways of data access to emerge, such as virtual realities (Deligiannidis and Jacob 2005, Laha et al. 2012), interactive access to virtual specimens on mobile devices (Johnson et al. 2012) or holographic representations (Javidi and Tajahuerce 2000, Midgley and Dunin-Borkowski 2009, Boistel et al. 2011).

Despite certain obvious advantages of 3D-imaging technologies, they will need time to evolve into a widely adopted method. At its core, taxonomy is a very traditional discipline, and commonly, changes are adopted at a slow pace. The aforementioned transition of taxonomy into a more data-centric and electronic discipline will need time so the community can learn how to make the best use of this new type of data and the information it contains, as well as to develop the necessary skills to handle these data. This goes hand in hand with technical obstacles that prevent the method from becoming widely used at the present: access to 3D imaging facilities is, although steadily increasing, still limited and often expensive. Special technical skills are needed to produce and process the data, and even with such expertise, the creation of the final dataset (the cybertype) is still a very time-consuming process. However, these arguments hold true for many new technologies, and it will be for the community to decide whether the information value contained in 3D-datasets will allow the technology to survive and to shape the future direction of taxonomy.

## Conclusions

Up to now, morphology-based systematics and taxonomy have not been able to keep pace with the rapid developments and data creation that characterise other disciplines. New technologies, such as micro-CT and other imaging techniques, will allow massive, computer-accessible data production. This will, in turn, inspire the development of new tools to manage and analyse these data, allowing large-scale morphology-based phylogenies, semi-automated identifications, the formulation of new systematic hypotheses, and advanced research on novel ways of managing, visualising and publishing data. The combined efforts of humans and new technologies will help the discipline to find its way into the digital age and might trigger its renaissance with an impact rivalling the discoveries of the great naturalist era of the 19^th^ century.
