# Supplementary figures and images for: Micro-computed tomography: Introducing new dimensions to taxonomy
Source: Zookeys. 2013 Feb 4;(263):1–45. doi: 10.3897/zookeys.263.4261 (PMC3591762; doi:10.3897/zookeys.263.4261)

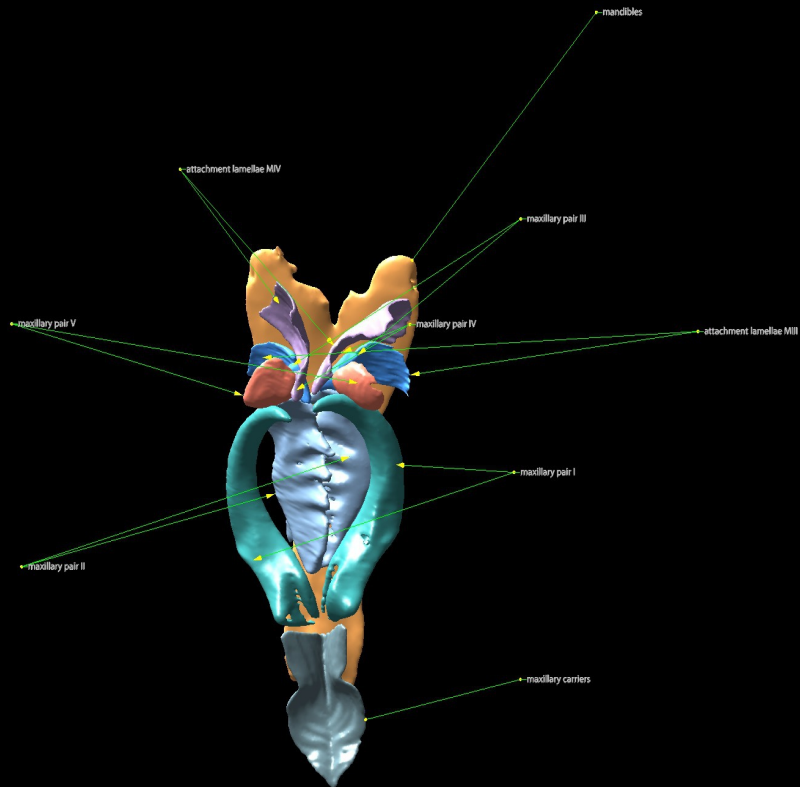

Supplement: Supplementary file 2 [file ZooKeys-263-001-g003.pdf]

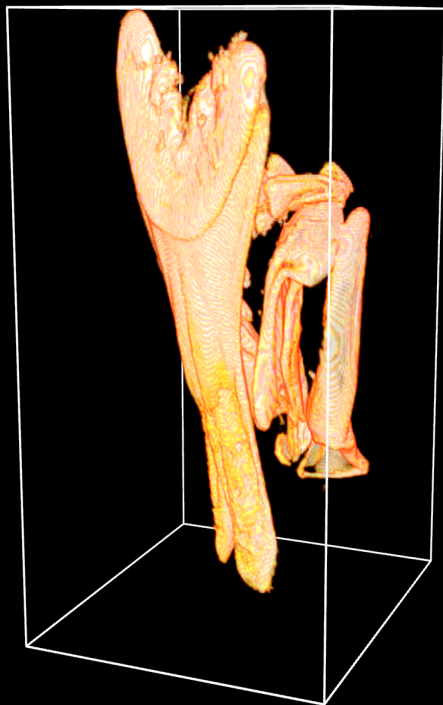

Supplement: Supplementary file 3 [file ZooKeys-263-001-g004.pdf]

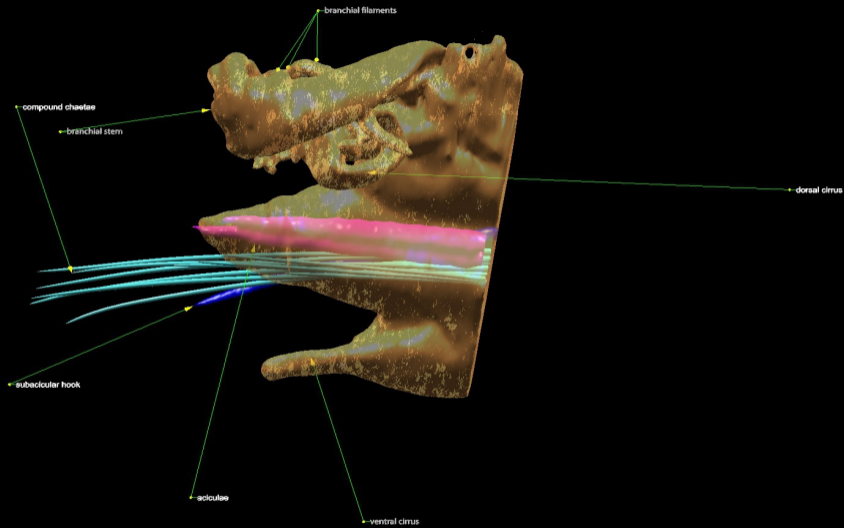

Supplement: Supplementary file 4 [file ZooKeys-263-001-g009.pdf]
